# Current cancer situation in China: good or bad news from the 2018 Global Cancer Statistics?

**DOI:** 10.1186/s40880-019-0368-6

**Published:** 2019-04-29

**Authors:** Rui-Mei Feng, Yi-Nan Zong, Su-Mei Cao, Rui-Hua Xu

**Affiliations:** 1 Department of Cancer Prevention Research Center, State Key Laboratory of Oncology in South China, Collaborative Innovation Center for Cancer Medicine, Sun Yat-Sen University Cancer Center, 651 Dongfeng Road East, Guangzhou, 510060 Guangdong P. R. China; 2 Department of Medical Oncology, State Key Laboratory of Oncology in South China, Collaborative Innovation Center for Cancer Medicine, Sun Yat-Sen University Cancer Center, 651 Dongfeng Road East, Guangzhou, 510060 Guangdong P. R. China

**Keywords:** GLOBCAN 2018, Cancer pattern, China, USA, UK, Carcinogenic risk factor, Tobacco smoking, Chronic infection, Westernized lifestyles, Cancer control strategies

## Abstract

Cancer is the leading cause of death in China and depicting the cancer pattern of China would provide basic knowhows on how to tackle it more effectively. In this study we have reviewed several reports of cancer burden, including the Global cancer statistics 2018 and Cancer statistics in China, 2015, along with the GLOBCAN 2018 online database, to investigate the differences of cancer patterns between China, the United States (USA) and the United Kingdom (UK). An estimated 4.3 million new cancer cases and 2.9 million new cancer deaths occurred in China in 2018. Compared to the USA and UK, China has lower cancer incidence but a 30% and 40% higher cancer mortality than the UK and USA, among which 36.4% of the cancer-related deaths were from the digestive tract cancers (stomach, liver, and esophagus cancer) and have relatively poorer prognoses. In comparison, the digestive cancer deaths only took up ≤ 5% of the total cancer deaths in either USA or UK. Other reasons for the higher mortality in China may be the low rate of early-stage cancers at diagnosis and non-uniformed clinical cancer treatment strategies performed by different regions. China is undergoing the cancer transition stage where the cancer spectrum is changing from developing country to developed country, with a rapidly increase cancer burden of colorectal, prostate, female breast cancers in addition to a high occurrence of infection-related and digestive cancers. The incidence of westernized lifestyle-related cancers in China (i.e. colorectal cancer, prostate, bladder cancer) has risen but the incidence of the digestive cancers has decreased from 2000 to 2011. An estimated 40% of the risk factors can be attributed to environmental and lifestyle factors either in China or other developed countries. Tobacco smoking is the single most important carcinogenic risk factor in China, contributing to ~ 24.5% of cancers in males. Chronic infection is another important preventable cancer contributor which is responsible for ~ 17% of cancers. Comprehensive prevention and control strategies in China should include effective tobacco-control policy, recommendations for healthier lifestyles, along with enlarging the coverage of effective screening, educating, and vaccination programs to better sensitize greater awareness control to the general public.

## Introduction

Cancer has been the leading cause of death worldwide. The situation in China has been alarming partly due to its rapid population growth and socioeconomic development. Recently, the International Agency for Research on Cancer (IARC) issued the worldwide cancer burden for 2018 based on the GLOBOCAN [1]. As to previous reports for 2002, 2007, 2008 and 2012, the report detailed cancer incidence and mortality at a global level, considering the geographic variability observed in 185 countries across 20 predefined world regions, commented on the associated risk factors and prospects for the prevention of major cancers observed worldwide.

Cancer patterns in China is an important focus of public health for several reasons. First, China is the most populous country in the world with an estimated population of nearly 1.42 billion, and by year 2020 to have around 4.51 million cancer cases and 3.04 million cancer deaths [2], which will undoubtedly impact the functional status, psychological well-being, and quality of life of its cancer sufferers and relatives. Second, due to the rapid social and economic development, cancer transitions are most highlighted in China, where an increasing amount of cancer is paralleled by a changing profile of common cancer types, which increases the difficulty for cancer control in the huge Chinese population as the control strategies differ considerably across cancer types and regions. Third, there are regions in China that are more prone for some specific types of cancers due to their local lifestyle habits, and shedding more light on these cancerous risk factors may help in improving the cancer burden. For instance, the Qidong region in Jiangsu province has a higher incidence of liver cancer which is possibly related to the high hepatitis B virus (HBV) infection rate and corns intake polluted by fungal aflatoxins because of weather condition and the way of the corns are stored in Qidong. The risk of nasopharyngeal carcinoma (NPC) in Southern China is very high and possibly related to the high consumption of salted-preserved fish and high prevalence of Epstein–Barr virus (EBV) infection. In the report of the GLOBCAN 2018, the cancer incidence and mortality in Western countries have shown a trending declined through decades of mountainous efforts in cancer prevention and control. However, the cancer burden in China during recent years is still, although stable, at a high level. Therefore, we reviewed the Global cancer statistics 2018 [1], Cancer statistics in China, 2015 [3], along with the GLOBCAN 2018 online database [2] to compare the difference of cancer burden, cancer type and cancer control strategies between China and the very high/high Human Development Index (HDI) countries of the Western world, such as the United States (USA) and United Kingdom (UK). By performing such a comparison and based on the observed differences, we hope that our finding would contribute for efforts to eliminate, at least, the avoidable cancer causes and to implement better cancer-prevention strategies in China.

### Age-standardized cancer incidence and mortality

It was estimated that there would be 18.1 million new cases (including nonmelanoma skin cancer, NMSC) and 9.6 million cancer deaths (including NMSC) worldwide in 2018, of which, nearly 24% (4.3 million) of these cancer cases and 30% (2.9 million) of deaths have occurred in China. The age-standardized cancer incidence in China (201.7/100,000) is comparable to that of the overall worldwide incidence (197.9/100,000) but lower than those in the UK (319.2/100,000) and USA (352.2/100,000) (Table 1). However, China had a much higher cancer mortality (130.1/100,000 for China vs. 102.6/100,000 for UK and 91.0/100,000 for USA) (Table 2), which might be due to the different cancer pattern by countries, lower early cancer detection rate and substandard treatment strategies provided by different regions in China. It is observed that 50% cases of digestive cancers, including stomach, liver, and esophagus cancer, occurred in China in 2018, and their 5-year overall survival rates were quite low, < 35% in 2013–2015 [4].

**Table 1:** The main 5 most commonly diagnosed cancer types and age-standardized incidence rate in 2018 in China, UK, USA, and worldwide. Source: GLOBOCAN [2]

China			UK			USA			Worldwide		
Tumor location	Cases (%)	ASR (worldwide, 1/10^5^)	Tumor location	Cases (%)	ASR (worldwide, 1/10^5^)	Tumor location	Cases (%)	ASR (worldwide, 1/10^5^)	Tumor location	Cases (%)	ASR (worldwide, 1/10^5^)
*Both sexes*											
Lung	774,323 (18.1)	35.1	Prostate	56,401 (12.6)	80.7	Breast	234,087 (11.0)	84.9	Lung	2,093,876 (11.6)	22.5
Colorectum	521,490 (12.2)	23.7	Breast	55,439 (12.4)	93.6	Lung	227,356 (10.7)	35.1	Breast	2,088,849 (11.6)	46.3
Stomach	456,124 (10.6)	20.7	Lung	52,320 (11.7)	32.5	Prostate	212,783 (10.0)	75.7	Colorectum	1,849,518 (10.2)	19.7
Liver	392,868 (9.2)	18.3	Colorectum	47,892 (10.7)	32.1	Colorectum	155,098 (7.3)	25.6	Prostate	1,276,106 (7.1)	29.3
Breast	367,900 (8.6)	36.1	Skin melanoma	17,852 (4.0)	15.0	Bladder	82,501 (3.9)	12.0	Stomach	1,033,701 (5.7)	11.1
All	4,285,033 (100.0)	201.7	All	446,942 (100.0)	319.2	All	2,129,118 (100.0)	352.2	All	18,078,957 (100.0)	197.9
*Male*											
Lung	518,547 (21.9)	47.8	Prostate	56,401 (23.6)	80.7	Prostate	212,783 (18.5)	75.7	Lung	1,368,524 (14.5)	31.5
Stomach	319,470 (13.5)	29.5	Lung	27,008 (11.3)	35.5	Lung	121,408 (10.6)	40.1	Prostate	1,276,106 (13.5)	29.3
Colorectum	303,853 (12.8)	28.1	Colorectum	26,551 (11.1)	37.8	Colorectum	80,829 (7.0)	28.8	Colorectum	1,026,215 (10.9)	23.6
Liver	292,898 (12.4)	27.6	Skin melanoma	9200 (3.8)	15.0	Bladder	63,263 (5.5)	20.0	Stomach	683,754 (7.2)	15.7
Esophagus	214,090 (9.0)	19.7	Bladder	8826 (3.7)	10.8	Skin melanoma	42,108 (3.7)	14.9	Liver	596,574 (6.3)	13.9
All	2,366,010 (100.0)	223.0	All	239,387 (100.0)	344.7	All	1,147,251 (100.0)	393.2	All	9,456,418 (100.0)	218.6
*Female*											
Breast	367,900 (19.2)	36.1	Breast	55,439 (26.7)	93.6	Breast	234,087 (23.8)	84.9	Breast	2,088,849 (24.2)	46.3
Lung	255,776 (13.3)	22.8	Lung	25,312 (12.2)	30.2	Lung	105,948 (10.8)	30.8	Colorectum	823,303 (9.5)	16.3
Colorectum	217,637 (11.3)	19.4	Colorectum	21,341 (10.3)	27.0	Colorectum	74,269 (7.6)	22.6	Lung	725,352 (8.4)	14.6
Thyroid	147,618 (7.7)	15.8	Corpus uteri	10,677 (5.1)	15.6	Corpus uteri	57,004 (5.8)	20.1	Cervix uteri	569,847 (6.6)	13.1
Stomach	136,654 (7.1)	12.3	Skin melanoma	8652 (4.2)	15.3	Thyroid	46,348 (4.7)	22.3	Thyroid	436,344 (5.1)	10.2
All	1,919,023 (100.0)	182.6	All	207,555 (100.0)	299.8	All	981,867 (100.0)	321.2	All	8,622,539 (100.0)	182.6

UK, the United Kingdom; USA, the United States of America; ASR, age-standardized rate

**Table 2:** The main 5 most common causes of cancer-related deaths and age-standardized mortality rate in 2018 in China, UK, USA and worldwide. Source: GLOBOCAN [2]

China			UK			USA			Worldwide		
Tumor location	Deaths (%)	ASR (worldwide, 1/10^5^)	Tumor location	Deaths (%)	ASR (worldwide, 1/10^5^)	Tumor location	Deaths (%)	ASR (worldwide, 1/10^5^)	Tumor location	Deaths (%)	ASR (worldwide, 1/10^5^)
*Both sexes*											
Lung	690,567 (24.1)	30.9	Lung	37,688 (21.1)	22.2	Lung	152,423 (24.7)	22.1	Lung	1,761,007 (18.4)	18.6
Stomach	390,182 (13.6)	17.5	Colorectum	20,957 (11.7)	11.1	Colorectum	54,611 (8.9)	8.2	Colorectum	880,792 (9.2)	8.9
Liver	368,960 (12.9)	17.1	Prostate	13,145 (7.4)	12.7	Pancreas	45,574 (7.4)	6.6	Stomach	782,685 (8.2)	8.2
Esophagus	283,433 (9.9)	12.7	Breast	11,849 (6.6)	14.4	Breast	41,904 (6.8)	12.7	Liver	781,631 (8.2)	8.5
Colorectum	247,563 (8.6)	10.9	Pancreas	10,043 (5.6)	6.0	Liver	30,485 (4.9)	4.9	Breast	626,679 (6.6)	13.0
All	2,865,174 (100.0)	130.1	All	178,473 (100.0)	102.6	All	616,714 (100.0)	91.0	All	9,555,027 (100.0)	101.1
*Male*											
Lung	472,142 (26.4)	43.4	Lung	19,918 (20.9)	25.2	Lung	81,307 (25.0)	25.9	Lung	1,184,947 (22.0)	27.1
Liver	273,014 (15.2)	25.6	Prostate	13,145 (13.8)	12.7	Prostate	28,705 (8.8)	7.7	Liver	548,375 (10.2)	12.7
Stomach	271,013 (15.1)	25.0	Colorectum	11,186 (11.7)	13.3	Colorectum	28,658 (8.8)	9.6	Stomach	513,555 (9.5)	11.7
Esophagus	197,823 (11.0)	18.2	Esophagus	5812 (6.1)	7.9	Pancreas	23,636 (7.3)	7.7	Colorectum	484,224 (9.0)	10.8
Colorectum	142,476 (8.0)	13.1	Pancreas	5022 (5.3)	6.7	Liver	20,564 (6.3)	7.2	Prostate	358,989 (6.7)	7.6
All	1,791,805 (100.0)	166.6	All	95,479 (100.0)	117.8	All	325,254 (100.0)	104.3	All	5,385,640 (100.0)	122.7
*Female*											
Lung	218,425 (20.3)	19.0	Lung	17,770 (21.4)	19.7	Lung	71,116 (24.4)	19.0	Breast	626,679 (15.0)	13.0
Stomach	119,169 (11.1)	10.4	Breast	11,849 (14.3)	14.4	Breast	41,904 (14.4)	12.7	Lung	576,060 (13.8)	11.2
Colorectum	105,087 (9.8)	8.8	Colorectum	9771 (11.8)	9.3	Colorectum	25,953 (8.9)	6.9	Colorectum	396,568 (9.5)	7.2
Breast	97,972 (9.1)	8.8	Pancreas	5021 (6.0)	5.3	Pancreas	21,938 (7.5)	5.6	Cervix uteri	311,365 (7.5)	6.9
Liver	95,946 (8.9)	8.6	Ovary	4155 (5.0)	4.9	Ovary	14,008 (4.8)	4.1	Stomach	269,130 (6.5)	5.2
All	1,073,369 (100.0)	95.2	All	82,994 (100.0)	90.6	All	291,460 (100.0)	80.3	All	4,169,387 (100.0)	83.1

UK, the United Kingdom; USA, the United States of America; ASR, age-standardized rate

### Main five cancer types

The most commonly diagnosed cancers in Chinese male, in 2018, were dominated by lung (21.9% of total cases), stomach (13.5%), colorectum (12.8%), liver (12.4%) and esophageal (9.0%) cancer, and for Chinese female they were breast (19.2% of total cases), lung (13.3%), colorectum (11.3%), thyroid (7.7%) and stomach (7.1%) cancer. For both sexes combined, China had comparable number of commonly diagnosed cancer cases, with lung, colorectum and female breast cancer (38.9% of total cases) as the UK (34.8%) and USA (29.0%), but had greater proportion of infection-attributable cancers (17.8% of liver and stomach cancer) (Table 1).

Digestive tract cancers, arising mainly from the stomach (13.6%), liver (12.9%) and esophagus (9.9%), were responsible for 36.4% of cancer-related deaths in China, which was only ≤ 5% of the total cancer deaths in either the USA or UK. For Chinese males, the 5 most common causes of cancer-related deaths were lung (26.4% of the total cancer deaths), liver (15.2%), stomach (15.1%), esophageal (11.0%) and colorectal cancer (8.0%); whereas among females they were lung (20.3% of the total cancer deaths), stomach (11.1%), colorectum (9.8%), breast (9.1%) and liver (8.9%) cancer. China is undergoing a transition period from being a developing country to a developed country and in regard to the commonly diagnosed cancer types, this is paralleling to a rapid cancer burden increase of colorectal, prostate, female breast cancers in addition to a high occurrence of infection-related and digestive cancers.

In comparison to UK and USA, China has a greater proportion of cancers having relatively poorer 5-year overall survival rate. For instance, although the incidence of lung cancer (~ 35/100,000) was similar between China and USA (Table 1), however, the mortality rate of lung cancer in China was 1.4 times greater than that of USA (Table 2). For female breast and colorectal cancers, the incidence rates in UK were 2.6 times and 1.4 times greater than that of China (Table 1), but our findings showed that the mortality rate in UK has decreased by 1.6 times and 0.9 times as compared to China, respectively (Table 2).

### Time trends of cancer incidence and mortality

According to the 2015 Chinese Cancer Statistics, the overall age-standardized cancer incidence and mortality in China were stable between 2000 and 2011 [3] but an obvious heterogeneity in time trends during the same time period was observed for the main cancers or most commonly diagnosed cancers. The incidence and mortality of colorectal cancer in male and female increased, but that of the esophageal, stomach, and liver cancer decreased. Meanwhile, the incidence rates of “westernized lifestyle-related cancer”, namely the prostate and bladder cancer in males, together with obesity and hormonal exposure-related cancers, namely thyroid, breast, and ovarian cancer in females showed an upward trend [3].

**Fig. 1:**
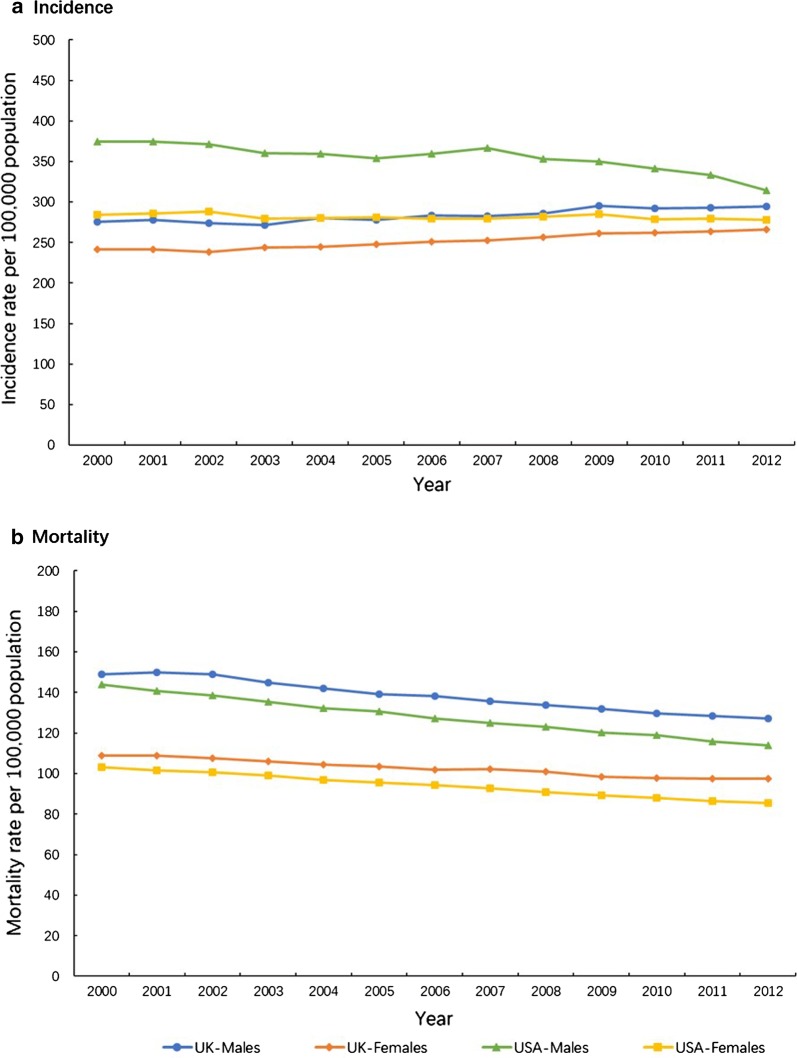
Trends in the age-standardized cancer incidence rate (**a**) and mortality rate (**b**) in the UK and USA by sex, between 2000 and 2012. UK, the United Kingdom; USA, the United States of America. Source: CI5*plus*; WHO cancer mortality database

**Fig. 2:**
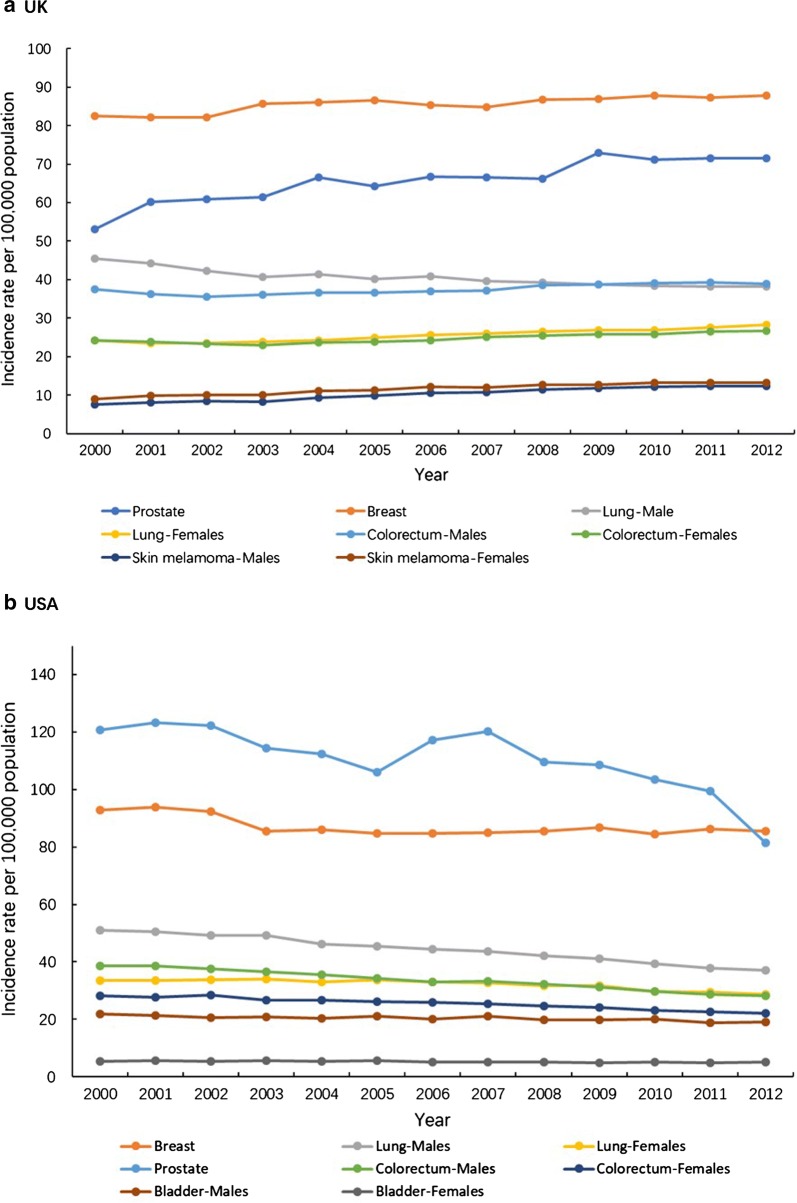
Trends in the age-standardized incidence rate of the top 5 incident cancer type in the UK (**a**) and USA (**b**), between 2000 to 2012. UK, the United Kingdom; USA, the United States of America. Source: CI5*plus* database

**Fig. 3:**
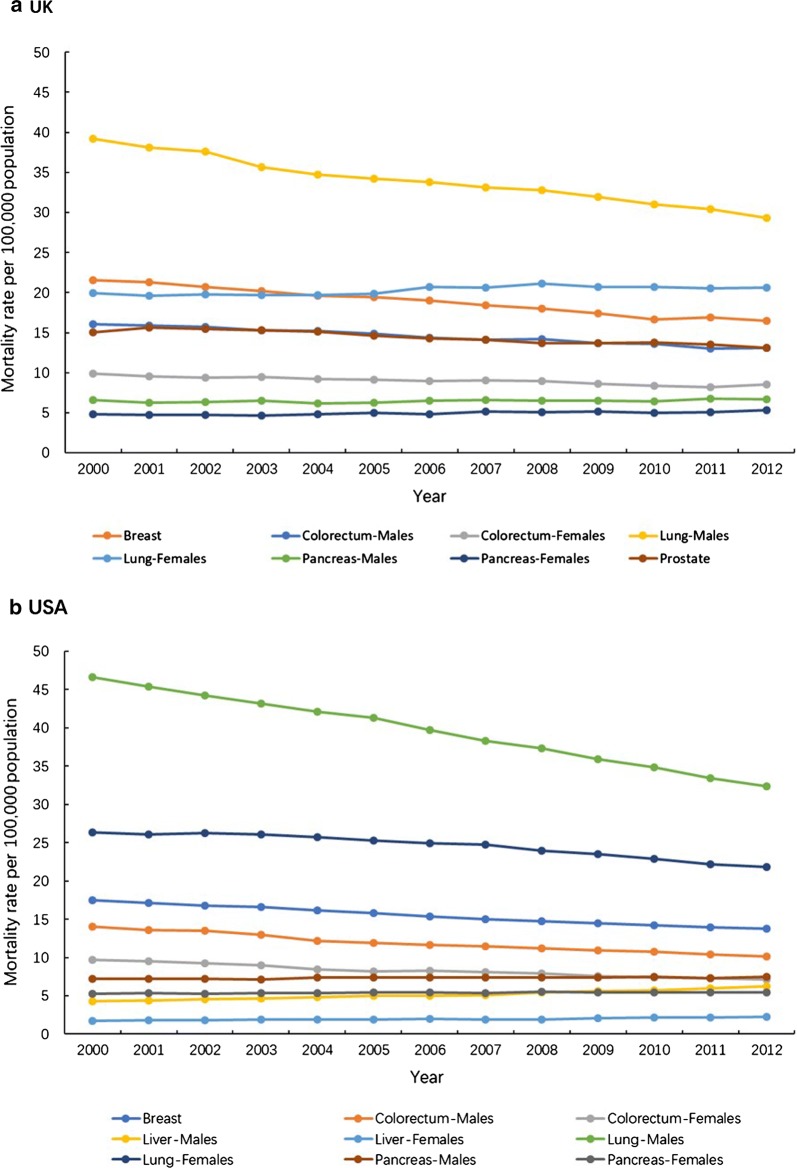
Trends in the age-standardized mortality rate of the top 5 mortal cancer in the UK (**a**) and USA (**b**), between 2000 and 2012. UK, the United Kingdom; USA, the United States of America. Source: WHO cancer mortality database

In contrast, between 2000 and 2012 the overall cancer incidence and mortality rate of all cancer-types in USA showed an obvious downward trend (Fig. 1) [5, 6]. However, despite the incidence rate in the UK increased slightly from 2000 to 2012 (Figs. 1a, 2a), the overall cancer mortality rate of all cancer-types has declined by ~ 11% in females and ~ 15% in males (Fig. 1b) [5], with simultaneous decrease in male mortality rate of lung, colorectal and prostate cancer by 25%, 18% and 13%, respectively, while the female mortality rate of breast cancer and colorectal cancer has decreased by 24% and 13%, respectively, and female pancreas and lung cancer mortality rate has increased by 9% and 3% (Fig. 3a) [6].

The all cancer-types incidence and mortality rate in USA between 2000 and 2012 have declined more rapidly in males (16% and 21%) than in females (2% and 17%) (Fig. 1) [5, 6]. The mortality rate of lung and colorectal cancer in males have decreased by 33% and 27%, and the mortality rate of breast, lung and colorectum cancer in females decreased by 21%, 17% and 27% [1] (Fig. 3b) [6].

### Main risk factors for cancer and primary prevention

It has been estimated that about 40% of risk factors are attributed to environmental and lifestyle conditions which can be preventable in both China or in other developed countries [7–9] (Table 3). However, the risk attributable-fraction contributing to individual cancer risk differ largely between countries (Table 3).

**Table 3:** The proportion of incident cancer cases attributable to risk factors by sex in China, UK, and USA

Risk factors^a^	Population attributable fraction (%)		
	China (2013) [8]	UK (2015) [7]	USA (2014) [9]
*Both sexes*			
All risk factors	38.5	37.7	42.0
Smoking	14.8	15.1	19.0
Second-hand smoking	1.9	NA	0.4
High BMI	2.9	6.3	7.8
Alcohol	2.9	3.3	5.6
Ultraviolet radiation	NA	3.8	4.7
Physical inactivity	0.9	0.5	2.9
Low fruit and vegetable consumption	5.7	NA	1.9
Low fiber	NA	3.3	0.9
Processed meat	NA	1.5	0.8
Infection	16.7	3.6	3.3
HBV infection	7.2	NA	0.1
HCV infection	1.2	NA	0.4
HPV infection	2.9	NA	1.8
*H. pylori* infection	4.8	NA	0.5
*Males*			
All risk factors	47.3	38.6	42.5
Smoking	24.5	17.7	23.6
Second-hand smoking	0.7	NA	0.4
High BMI	2.7	5.2	4.8
Alcohol	4.8	3.1	4.8
Ultraviolet radiation	NA	3.8	5.8
Physical inactivity	0.5	0.5	1.5
Low fruit and vegetable consumption	6.8	NA	2.2
Low fiber	NA	3.1	0.9
Processed meat	NA	2.1	1.1
Infection	17.7	3.1	3.3
HBV infection	9.5	NA	0.1
HCV infection	1.5	NA	0.8
HPV infection	0.2	NA	1.2
*H. pylori* infection	5.7	NA	0.4
*Females*			
All risk factors	27.8	36.8	41.5
Smoking	2.4	12.4	14.5
Second-hand smoking	3.4	NA	0.3
High BMI	3.1	7.5	10.9
Alcohol	0.5	3.5	6.4
Ultraviolet radiation	NA	3.7	3.7
Physical inactivity	1.4	0.5	4.4
Low fruit and vegetable consumption	4.2	NA	1.5
Low fiber	NA	3.4	1.0
Processed meat	NA	0.9	0.5
Infection	15.4	4.2	3.3
HBV infection	4.3	NA	0.1
HCV infection	0.7	NA	0.1
HPV infection	6.3	NA	2.5
*H. pylori* infection	3.6	NA	0.5

*UK*, the United Kingdom; *USA*, the United States of America; *BMI*, body mass index; *HBV*, hepatitis B virus; *HCV*, hepatitis C virus; HPV, human papillomavirus; *H. pylori*, *Helicobacter pylori*

^a^Some risk factors are not shown in table due to some data was not available and indicated by NA

Tobacco is the leading cause of cancer worldwide, attributed to the development of ~ 20 malignancies and primarily responsible for > 70% of the worldwide lung cancer cases. Further, it is estimated that 24.5%, 23.6% and 17.7% of male cancers in China, USA, and UK, respectively are due to tobacco smoking [8]. The smoking rate (~ 3% [10]) in Chinese females is comparatively lower (USA [11], 15.3%; UK [12], 20%) but is still contributing to ~ 2.4% of female cancers [8]. In contrast, the fraction attributable to tobacco smoking for female cancers was 14.5% [9] and 12.4% [7] in USA and UK. However, it has been found that more females in China are exposed to secondhand smoking, with an attribution risk for cancer of 3.4% [8], and as compared to 0.3% in USA [9].

Due to the harmful consequences of the tobacco epidemic on the public health, the World Health Organization (WHO) issued the Framework Convention on Tobacco Control in 2003 with the aim to reduce the global cigarette consumption rate. Based on this, 168 countries signed the agreement and drafted their own tobacco control strategies. USA was the first to implement a strict tobacco control campaign since the 1960s, which has consequently shown a continuous decrease in the number of smokers which has dropped from 42% in 1965 to 14% in 2017 [13]. As such, the lung cancer mortality rate in males from USA decreased by 43% from 1990 to 2014. In this regard, UK adopted the most powerful measures for tobacco control and found a reduction in smoking rate by nearly 50% from the 1970s to 15% in 2016. The lung cancer incidence fell by more than 25% and the mortality rate has been in constant decline, dropping by 35% from 1995 to 2013 [13]. However, the tobacco control situation in China is still unsatisfactory. Based on the 2015 China Tobacco Control Report, the adult male smoking rate was found to be > 50%, which parallel with the persistently high incidence rate of lung cancer in males of China. Meanwhile, the incidence and mortality of lung cancer in females in China may be more likely contributed to second-hand smoking exposure or occupational related risk factors.

The largest preventable cancer-contributor in China is chronic infection, which is responsible for about 17% of all cancers in China [8] and is predominantly comprised of *H. pylori* (stomach cancer), HBV (liver cancer), human papillomavirus (HPV; cervical cancer), and EBV (NPC). Comparatively, only < 4% of the cancers in USA or UK are attributed to these chronic infections.

The most effective strategy for the prevention of infection-related cancers is to create more effective vaccines against these carcinogenic viruses and to formulate better annihilation ways to combat these bacteria. In China, more than 78% of liver cancers are caused by chronic infection [8]. The recent decline of incidence and mortality rate of liver cancer in China has been partly attributed to infant routine immunization against HBV, implemented since 1992. The hepatitis B antigen (HBsAg) prevalence has since decreased by 90% among children aged between 1 and 4 years, and by 86% and 72% among those aged 5–9 and 10–14 years, respectively [14].

The proven biological etiology of cervical cancer is HPV infection, among which HPV16 and HPV18 are attributed to > 70% of the worldwide cervical cancer [15]. HPV vaccination was first introduced in America [16] in 2006 and since then the vaccinated-type HPV prevalence has decreased from 11.5% in the pre-vaccinated era (2003–2006) to 5.1% in the vaccinated era (2007–2010) among females aged between 14 and 19 years old [17]. The HPV vaccination in China has lagged for more than 10 years until 2017 when one bivalent vaccine was approved by China Food and Drug Administration but this is yet to be included in the national immunization schedules.

EBV infection is a known risk factor related to the development of NPC. It is responsible for ~ 30% of the NPC cases and ~ 40% of the NPC-related deaths worldwide emerging from Southern China [2]. However, until now there is still no vaccine against the EBV infection to combat NPC.

Chronic infection with *H. pylori* is the strongest risk factor for gastric cancer. China has higher *H. pylori* prevalence (56%) as compared to UK and USA (35.5% for UK and 35.6% for USA) [18], which may be responsible to the low incidence of gastric cancer in UK and USA (< 5 per 100,000). In Chinese males, the incidence of stomach cancer has declined by 5.3% per year from 2000 to 2003, and 1.8% from 2003 to 2011 and a decrease in mortality rate of stomach cancer has also been observed, 7.5% per year from 2000 to 2003, and 2.3% from 2003 to 2011 and similar decline in incidence and mortality rate has also been observed among Chinese females [3]. The reasons for these declines are complex and not well understood but are thought to be partly caused by the decrease in *H. pylori* prevalence as a result of wider population-based screening and greater awareness for treating *H. pylori* infection.

Although not exactly elucidated, dietary and lifestyle components are also likely to be of major importance for the high-frequency cancers observed in China. The intake food with low nutrition or contamination with nitrosamines might be a key factor for the high prevalence of upper digestive tract cancers. The decline in the incidence rate of esophageal and stomach cancers in China by 1.8%–5.5% per year from 2000 to 2011 [3], is believed to be due to improved hygiene, better food preservation using refrigeration and consumption of less salty but more nutritious foods such as fruits and vegetables. Meanwhile, the undergoing westernized lifestyle transition in China had contributed to the rise in several cancers including pancreatic, colorectal, prostate, and female breast cancers, which may be influenced by the high consumption of red/processed meats, increase in obesity and sedentary lifestyle among the Chinese population.

### Cancer screening for early cancer detection

The implementation of population-based screening program can significantly reduce the mortality and incidence of cancer, particularly that of breast, cervical and colorectal cancers. The UK has implemented a national population-based cancer screening for breast and cervical cancer since 1980s, and one for bowel cancer since 2006 [19, 20], which has led to a reduction in cancer burdens, correspondingly, the mortality rate has dropped from 28.92 to 15.90 per 100,000 for female breast cancer and from 4.78 to 1.64 per 100,000 for cervical cancer during 1990–2013; the mortality rate of colorectum cancer has decreased from 14.36 to 12.67 per 100,000 in males and from 8.97 to 8.26 per 100,000 in females during 2006–2013 [6]. Achieving the high population coverage and adherence for the target population with appropriate screening methods are critical to the screening effectiveness. It has been found that the adherence rates in screening programs were relatively high in USA, with an adherence rate of 71.5%, 83%, and 62% for screening breast, cervical and colorectal cancer, respectively [21]. Several European countries have also started full coverage for these three cancer screening since 2007, and the actual screening rate of the European Union for cervical cancer is 72%, and breast cancer is 95% by 2016 [21].

Although until now there is no national scale cancer screening in China, several population-based cancer screenings supported by the government have been implemented in high-risk areas for, stomach, esophageal, colorectal, liver, lung, nasopharyngeal and female breast cancer since 2006. Besides, spontaneous cancer screening in urban areas is becoming more popular, despite those being self-paid. These screening programs may partly explain for the declines in incidence and mortality of stomach and esophagus cancer in China. The main barrier for cancer screening in China is that the population coverage for the entire country is still insufficient and needs further improvement. For instance, the current largest female breast screening program has a coverage reaching ~ 50% of its targeted population despite its implementation since 2009.

Screening for cancer has many benefits, but not without potential risks. Not all precancerous lesions or cancers detected on screening may become symptomatic or life-threatening. The risk for overdiagnosis can lead to overtreatment and cause more harm than gain. The rapidly increasing incidence and relatively stable mortality of thyroid cancer and prostate cancer in many countries including China, USA, and UK in recent decades might be heavily influenced by the over-diagnosis with ultrasonic or PSA testing for screening these two cancers. As such, in 2012, the US Preventive Services Task Force has recommended the cancellation of regular screening using the PSA test which has to some extent led to a decrease in prostate cancer incidence. However, the incidence rate of prostate cancer is still on the rise in some countries where PSA testing is widely used such as in UK and China.

### Cancer control strategy for the current cancer burden in China

Although cancer incidence in China is generally low compared to that of UK and USA, the cancer burden is still expected to rise in the following years because of the aging and growing population and the rise in westernized lifestyle. Correspondingly, the trend of cancer incidence in China has seen a rapid increased in the burden of colorectal, prostate, female breast cancers which also occur in developed countries such as in USA and UK, and a persistently heavy burden of digestive cancer or infection-related cancers such as liver cancer and stomach cancer, which often occur in the less developed countries. The relative laggard cancer control strategy in China can worsen this situation, therefore, the national cancer control program implemented by the government should be adjusted based on the best practices or cancer control strategies used for some cancers which have been evidence-based and well-established in the developed countries, and at the same time, taking consideration of the diversity of cancer types by of different regions in China, including the immense scale of population, the priorities for cancer burden by regions and the availability and access to medical resources.

In conclusion, effective strategies for decreasing the cancer burden in China should adopt comprehensive prevention and control measures. More public awareness and training are urgently needed to narrow the enlarging gap between advanced evidence-based knowledge of cancer prevention to reduce the established risk factors, especially in the less developed regions, which has been convinced to be the most potential cost-effective measure during long-term cancer control. Effective tobacco-control policies should be drafted or become more stringent and more focus should be placed on healthy lifestyles. In addition, we also recommend enlarging the coverage of effective screening, educating, and vaccination programs. After all, prevention is better than cure.

## Abbreviations

IARC: International Agency for Research on Cancer
HDI: Human Development Index
USA: the United States
UK: the United Kingdom
WHO: World Health Organization
*H. pylori*: *Helicobacter pylori*
HBV: hepatitis B virus
HPV: human papillomavirus
EBV: Epstein–Barr virus
NPC: nasopharyngeal carcinoma
NMSC: nonmelanoma skin cancer
BMI: body mass index
